# The Comparisons of Cerebral Hemodynamics Induced by Obstructive Sleep Apnea with Arousal and Periodic Limb Movement with Arousal: A Pilot NIRS Study

**DOI:** 10.3389/fnins.2016.00403

**Published:** 2016-08-31

**Authors:** Zhongxing Zhang, Maja Schneider, Marco Laures, Ming Qi, Ramin Khatami

**Affiliations:** ^1^Center for Sleep Medicine and Sleep Research, Clinic BarmelweidBarmelweid, Switzerland; ^2^Bern Network for Epilepsy, Sleep and Consciousness (BENESCO), Department of Neurology, University Hospital Bern, University of BernBern, Switzerland

**Keywords:** cerebral hemodynamics, sleep apnea, periodic limb movement during sleep, heart rate, near-infrared spectroscopy

## Abstract

Obstructive sleep apnea syndrome (OSA) and restless legs syndrome (RLS) with periodic limb movement during sleep (PLMS) are two sleep disorders characterized by repetitive respiratory or movement events associated with cortical arousals. We compared the cerebral hemodynamic changes linked to periodic apneas/hypopneas with arousals (AHA) in four OSA-patients with periodic limb movements (PLMA) with arousals in four patients with RLS-PLMS using near-infrared spectroscopy (NIRS). AHA induced homogenous pattern of periodic fluctuations in oxygenated (HbO_2_) and deoxygenated (HHb) hemoglobin, i.e., the decrease of HbO_2_ was accompanied by an increase of HHb during the respiratory event and resolved to reverse pattern when cortical arousal started. Blood volume (BV) showed the same pattern as HHb but with relative smaller amplitude in most of the AHA events_._These changing patterns were significant as Wilcoxon signed-rank tests gave *p* < 0.001 when comparing the area under the curve of these hemodynamic parameters to zero. By contrast, in PLMA limb movements induced periodic increments in HbO_2_ and BV (Wilcoxon signed-rank tests, *p* < 0.001), but HHb changed more heterogeneously even during the events coming from the same patient. Heart rate (HR) also showed different patterns between AHA and PLMA. It significantly decreased during the respiratory event (Wilcoxon signed-rank test, *p* < 0.001) and then increased after the occurrence of cortical arousal (Wilcoxon signed-rank test, *p* < 0.001); while in PLMA HR first increased preceding the occurrence of cortical arousal (Wilcoxon signed-rank test, *p* < 0.001) and then decreased. The results of this preliminary study show that both AHA and PLMA induce changes in cerebral hemodynamics. The occurrence of cortical arousal is accompanied by increased HR in both events, but by different BV changes (i.e., decreased/increased BV in AHA/PLMA, respectively). HR changes may partially account for the increased cerebral hemodynamics during PLMA; whereas in AHA probable vasodilatation mediated by hypoxia/hypercapnia is more crucial for the post-arousal hemodynamics. The differences between changes of cerebral hemodynamics and HR may indicate different pathological mechanisms behind these two sleep disorder events.

## Introduction

Obstructive sleep apnea syndrome (OSA) and restless legs syndrome (RLS) with periodic limb movement during sleep (PLMS) are two of the most common sleep disorders. OSA is characterized by periodic reduction (hypopnea) or cessation (apnea) of breathing due to narrowing or occlusion of the upper airway during sleep (Afzelius, 1981; Strollo and Rogers, 1996). The main features of PLMS are periodic, short-lasting and involuntary movements of the limbs during sleep, usually occurring in the legs and often associated with a partial arousal or awakening (Manconi et al., 2014). Patients with PLMS are usually unaware of their nocturnal movements and associated sleep disruption. OSA and PLMS usually share common features, such as the repetitive occurrence of respiratory and limb movement events (apnea phenomenon is characterized by a periodicity of 30–40 s, and PLMS' periodicity is 20–40 s), both are variably associated with cortical electroencephalogram (EEG) changes (i.e., cortical arousals; Ferrillo et al., 2004; Kohler and Stradling, 2010). Many patients with OSA or PLMS suffer from daytime sleepiness and fatigue. OSA is a well-known high risk factor of cardiovascular diseases including hypertension, ischemic heart disease, and stroke (Somers, 2005; Yaggi et al., 2005; Sharma, 2013); meanwhile a number of studies indicate that PLMS may also increase the risk for cardiovascular diseases although the pathological mechanisms are not yet definitive (Walters and Rye, 2009; Cuellar, 2013; Pennestri et al., 2013). Besides, PLMS is a common finding in patients with OSA.

Although both PLMS and OSA events are usually associated with cortical arousals, some differences exist. Arousals associated with OSA occur at the end of apnea and hypopnea events (AH), which may play a protective role to shortly wake up the brain to terminate the long-lasting hypoxia induced by AH. Arousal-induced reflex sympathetic activation can result in repetitive blood pressure and heart rate (HR) rises (Andreas et al., 1992; Yang et al., 2006), and may finally lead to endothelial dysfunction and cardiovascular diseases (Kohler and Stradling, 2010). By contrast in PLMS the occurrence of arousal is more heterogeneous, i.e., cortical arousal can occur just before the start of limb movements or after the limb movements (Ferrillo et al., 2004; Ferri et al., 2007, 2015) rendering the cause/effect relationship between the periodic limb movement (PLM) and arousal inconclusive. The PLMS may involve similar autonomic mechanisms (Manconi et al., 2012; Ferri et al., 2015) because PLM induced arousals (PLMA) are accompanied by pronounced blood pressure and HR increases (Ferrillo et al., 2004; Ferri et al., 2007; Guggisberg et al., 2007; Siddiqui et al., 2007; Pennestri et al., 2013).

As both AH with arousal (AHA) and PLMA may share common autonomic mechanisms to induce cortical arousals, we can assume that both events may affect cerebral hemodynamics. This assumption has important implications for both basic research and clinical treatment for several reasons. The similar periodicity and autonomic activation of PLMA and AHA lead to the hypothesis of a common central generator underlying OSA and PLMS (Carelli et al., 1999). However, no direct evidence of such a generator has been provided so far and recent studies demonstrating that PLM can be temporally dissociated from arousals (Manconi et al., 2012) challenged the hypothesis of a common generator. Clinical data from patients with co-occurring PLMS and AH support this hypothesis. These patients may present with PLMS after AH has been successfully treated by nasal continuous positive airway pressure (CPAP). Further increase of CPAP pressure eliminates residual PLM suggesting that insufficient CPAP pressure may cause PLMS in OSA-patients and PLMS were mainly respiratory driven (Baran et al., 2003; Seo and Guilleminault, 2012). Thus, further research on the causative and temporal link between cerebral hemodynamic changes and arousals induced by PLM/AH will add a valuable contribution to the discussion.

Several well-established neuroimaging methodologies including functional magnetic resonance imaging (fMRI), positron emission tomography (PET), and single-photon emission computed tomography (SPECT) are not suitable to study cerebral hemodynamics during naturally nocturnal sleep due to obvious reasons including safety issues, radiation, high magnetic fields, loud noises, motion artifacts, compatibility of sleep polysomnography (PSG) device, etc. Compared to these techniques, as a totally non-invasive and non-radiation optical method near-infrared spectroscopy (NIRS) can measure changes oxygenated (HbO_2_) and deoxygenated (HHb) hemoglobin (Hb) and total hemoglobin (tHb) in local cerebral regions with less restriction (Scholkmann et al., 2014; Zhang and Khatami, 2015). The nature of multi-variable measurement of NIRS makes it a useful tool to characterize the hemodynamics from the angles of both perfusion (e.g., changes in tHb) and oxygenation (e.g., changes in HbO_2_ and HHb). Several NIRS studies have been conducted to investigate cerebral hemodynamics in healthy population during all-night sleep (Hoshi et al., 1994; Pierro et al., 2012; Zhang and Khatami, 2014, 2015), and in patients with OSA (Hayakawa et al., 1996; Pizza et al., 2010; Ulrich et al., 2014; Zhang et al., 2014). All these studies in patients with OSA reported a cerebral hemodynamic pattern induced by AH events, i.e., HHb increases while HbO_2_ decreases during AH. There is only a single study describing cerebral hemodynamics in the prefrontal cortex in PLMS in a small number of heterogeneous patients (Pizza et al., 2009). But the various cardiovascular comorbidities in these patients make it difficult to interpret the cerebral hemodynamic changes because their cardiac function or blood pressure is abnormal and might influence cerebral hemodynamics.

In this study, we directly compare cerebral hemodynamic changes in HbO_2_, HHb, and tHb induced by AHA and PLMA with NIRS for the first time. Our special interest is on changes of tHb as a surrogate marker of blood volume (BV; Scholkmann et al., 2014; Ulrich et al., 2014) because increased or decreased BV could reflect vasodilatation or vasoconstriction. Our study will thus provide different aspects of hemodynamics including cerebral perfusion, oxygenation, and vasomotor activities. Given the similarities of aforementioned systemic hemodynamic parameters (i.e., blood pressure and HR changes) in AHA and PLMA our main hypothesis is that arousals in both AHA und PLM induce changes in cerebral hemodynamics. Our second hypothesis is that despite similar systemic hemodynamic changes the cerebral BV may change differently, as AH compared to PLM will result in stronger vasodilatation before the occurrence of arousal because respiratory events are accompanied with hypoxia and hypercapnia.

## Materials and methods

### Patients

Eleven patients were recruited for this pilot study. But three recordings were failed due to the falling down of NIRS sensors during sleep. The basic information of the other eight patients is shown in Table 1. PLMS usually occurs with other sleep disorders like RLS and it is frequently found in patients with OSA. In this study all our patients met the clinical criteria of OSA [i.e., apnea–hypopnea index (AHI) >4/h], but the patients with PLMS were all clinically diagnosed as RLS and had clear PLMS periods without any respiratory events. AHI is an index used to indicate the severity of sleep apnea. Its value is the number of apnea and hypopnea events per hour scored from PSG. As shown in Table 1, Patient No. 1–4 had been clinically diagnosed as PLMS and mild (AHI between 5 and 14) or moderate (AHI between 15 and 29) OSA, whereas patient No. 5–8 only had severe (AHI equals to or larger than 30) OSA. Patient No. 8 was recorded for the first 2 h of sleep and then it was decided to start CPAP therapy considering the severity of his OSA (AHI = 134/h). None of the OSA patients had any other cardiovascular diseases except for Patient No. 1 and No. 3 who had arterial hypertension. All the patients were recruited at Center for Sleep Medicine and Sleep Research, Clinic Barmelweid, Switzerland. The study was proven by the Aargau cantonal ethics committee, and all subjects gave their written informed consent to participate into the study.

**Table 1:** **Basic information of 8 patients**.

**Patient No**.	**Sex**	**Age (yrs)**	**BMI (kg/m^2^)**	**AHI (/h)**	**PLM index (/h)**	**Arousal index (/h)**
1	M	55	22.1	26	39	37
2	M	45	25.7	6	26	84
3	M	61	30.2	15	17	32
4	M	69	27.7	22	40	32
5	M	31	44	66	3	69
6	M	48	27.7	51	3	28
7	F	55	32.8	40	0	36
8	M	36	41.8	134	0	33

*BMI, body mass index; AHI, apnea-hypopnea index; PLM, periodic limb movement*.

### Video-polysomnography (PSG) measurements

A standard all-night Video-PSG (Embla RemLogic, Embla Systems LLC, Tonawanda, NY, USA) measurement was recorded from each patient. PSG is a multi-parametric test routinely used in sleep studies to assess clinical diagnosis of sleep disorders. It is a comprehensive recording of the biophysiological signals during sleep, including EEG at electrode locations of C3, C4, O1, O2, F3, and F4 according to 10–20 system, eye movements (electrooculogram, EOG), muscle activation (electromyogram, EMG), electrocardiogram (ECG), breathing functions (respiratory airflow and respiratory effort indicators), HR and peripheral oxygen saturation (SpO_2_). Each patient was videotaped with an infrared camera to allow for subsequent assessment of subject's movement during sleep. Two experienced neurophysiologist independently scored the sleep stages, respiratory and limb movement events, and motion artifacts in 30-s epochs according to the current version of American Academy of Sleep Medicine (AASM; Berry et al., 2014) based on the PSG measurements. We only scored leg movement for PLMA event, no upper limb movements were included in our analysis (in order to exclude any potential movement artifacts in NIRS signals induced by upper limb movements). Significant leg movement is defined as a 0.5–10 s period where EMG activity recorded by same configuration from the left or right anterior tibialis exceeds 8 μV above resting EMG and then falls below 2 μV from baseline for 0.5 s or longer. Then the differences between these two independent score results were further corrected by these two clinicians working together.

### NIRS measurements

A commercialized NIRS device (NIRO-300, Hamamatsu Photonics, Japan) was used to monitor the cerebral hemodynamic changes in this study (Suzuki et al., 1999). NIRO-300 used four wavelengths of near-infrared light (775, 810, 850, and 910 nm). The optical probe contained one light emitter and three closely placed sensors (i.e., an integrated three-segment photodiode chip with the separation of 1 mm). The distance between emitter and the center of the integrated photodiode chip was 4 cm [please refer to the literature (Suzuki et al., 1999) describing the design of NIRO-300]. NIRO-300 can locally measure relative changes in HbO_2_ and HHb by calculating the light attenuation changes in tissue based on modified Beer-Lambert law (MBLL; Al-Rawi et al., 2001; Scholkmann and Wolf, 2013; Scholkmann et al., 2014). The differential pathlength factor was 5.93 for all the wavelengths in adult head (van der Zee et al., 1992). The sum of changes in HbO_2_ and HHb was the changes in tHb, which reflect changes in BV. The DPF method can cause cross-talk between measured changes in HbO_2_ and HHb (Strangman et al., 2003). But it might be less of an issue in our study, as we mainly focused on changes in BV. In this study, the NIRO-300 probe was kept in contact with the left side of the subject's forehead, just below the hairline and above the left brow, via a medical adhesive and a soft black medical bandage. The sample rate of NIRS measurement was 1 Hz. The raw analog outputs (light intensities) of NIRO-300 measurements were sampled by the data acquisition system of video-PSG, so that NIRS and PSG measurements were synchronized.

### Data analysis

We focused on the comparison between the AHA and PLMA events because both events were associated with cortical arousal and autonomic activation. Therefore, we did not include any AH or PLM event without cortical arousal into analysis. Respiratory-related leg movements are also frequently found in patients with OSA, but this kind of leg movements is thought to be provoked by respiration and because of the periodic nature of OSA events they mimic PLMS (Manconi et al., 2014). We did not consider respiratory-related limb movements for analysis because the current criteria for the scoring of PLMS exclude those limb movements associated with respiratory events (Zucconi et al., 2006; Berry et al., 2014). Movement artifacts during Video-PSG can be recognized from EEG channels, and confirmed by visually checking the video recordings. Data associated with and within 3 min after movement artifacts were discarded. Fragments of NIRS signals that were without motion artifacts during repetitive (i.e., at least four consecutive events) AHA and PLMA were selected. All data were expressed as means (standard error) unless indicated otherwise.

#### Pre-processing of NIRS signals to remove physiological interferences

The fragments of NIRS signals in the same patient were first connected into a whole data sequence. Usually there are several “jumps” in this connected data sequence because of the discontinuity between the connected fragments, but they can be eliminated by correcting for the difference between the first point of the following fragment and the last point of the previous one (Zhang and Khatami, 2014). After that the NIRS signals were subjected to a low pass filter (Hanning Window with a cut-off frequency of 0.08 Hz) to remove the respiratory noise (0.2–0.3 Hz) and spontaneous slow hemodynamic oscillations (0.1–0.15 Hz); (Zhai et al., 2009). Then the filtered data were smoothed with moving average smooth method [i.e., robust locally weighted scatter plot smoothing (Cleveland and Devlin, 1988), which assigns zero weight to data outside six mean absolute deviations].

#### Area under the curve (AUC) of NIRS signals

AUC calculated as the integrals of changing curve is a parameter that can assess hemodynamic changes during a predefined period (i.e., positive value of AUC suggests increasing hemodynamic changes while negative one indicates decreasing trend) and it has been used in fMRI studies (Hirano et al., 2011), as well as in NIRS studies to analyse cerebral hemodynamic changes in sleep apnea events (Pizza et al., 2010). It is well-known that AHA and PLMA events usually vary in duration and last for several tens of seconds. AUC is a powerful tool for analyzing AHA and PLMA as the value of AUC does not only depend on the maximum value, but also on the duration of the event. Therefore, AUC is more suitable than to compare the peaks or nadirs of the signals within the periods (e.g., sometimes improper “peak” or “nadir” during long events may be induced by artifacts rather than true changes of the signals). Here we subjected changes in HbO_2_, HHb, and BV of AHA and PLMA events to AUC. First the NIRS signals of each AHA and PLMA event were extracted from the whole data sequence that after pre-processing. Then the slow baseline drifts of NIRS signals during each event were linearly detrended, and in each event the mean values of the first three data points were subtracted from the NIRS signals (i.e., baseline correction to align the starting points of integrals to be around 0). Finally the relative changes of NIRS signals in each event were integrated to calculate AUC. Please note that the AUC could be negative value in our calculation if the integrated curve below x-axis (Pizza et al., 2010). We did not take the absolute value of the integration, as our intention was to characterize the dynamics of NIRS signals (i.e., positive and negative values of AUC can indicate different changing directions).

#### Heart rate (HR) changes

HR was derived from PSG recordings by calculating the peak-to-peak pulsation interval of the measured photoplethysmographic waveform. In PLMA, the relative HR changes (with the HR value and the time of the onset of leg movement as reference value 0) within the time window from −5 to 25 s were averaged over all the PLMA events from all the patients. The time threshold boundary of −5 was arbitrary while the boundary of 25 after the onset of PLMA was derived from the peak of the histogram of the length of all events, i.e., the most frequent time length of PLMA events. Unlike the average of HR changes in PLMA events which only had one state transition (i.e., the starts of leg movement and arousal were almost synchronized), in AHA events there were two transition points (i.e., the start of AH and the start of arousal) and the time interval between them varied among the events. We cannot simply average all the events by aligning them to one time point. For example, if we chose the start of AH as reference time to average all the events, we cannot obtain the accurate information of HR changes at the start of arousals because of the variation of time intervals between these two time points. Therefore, we chose the HR at the start of AH as baseline and then quantified HR changes at the end of AH (i.e., the start of arousal) and 5 s after arousals, although this approach limits us to explore the changes in HR during the whole AHA events.

#### Statistical analysis

Shapiro-Wilk parametric test was performed to test the normality of the AUC of all NIRS parameters and HR changes. Parametric *t*-test (if the variables had normal distribution) or non-parametric Wilcoxon signed-rank tests (if the variables were not normal distribution) were used to compare whether the AUC of HbO_2_, HHb, and BV were significantly different from 0 in each patient and in each group of patients with OSA and PLMS (*p* < 0.05). Similarly, *t*-test or Wilcoxon signed-rank test was chosen to test whether the relative HR changes during PLMA and AHA were significant. All signal analysis and statistical analysis were carried out in MATLAB (The Math-Works, Inc., Natick, MA, USA).

## Results

### Typical patterns of cerebral hemodynamics in apnea/hypopnea with arousal (AHA) and periodic limb movement with arousal (PLMA)

**Figure 1:**
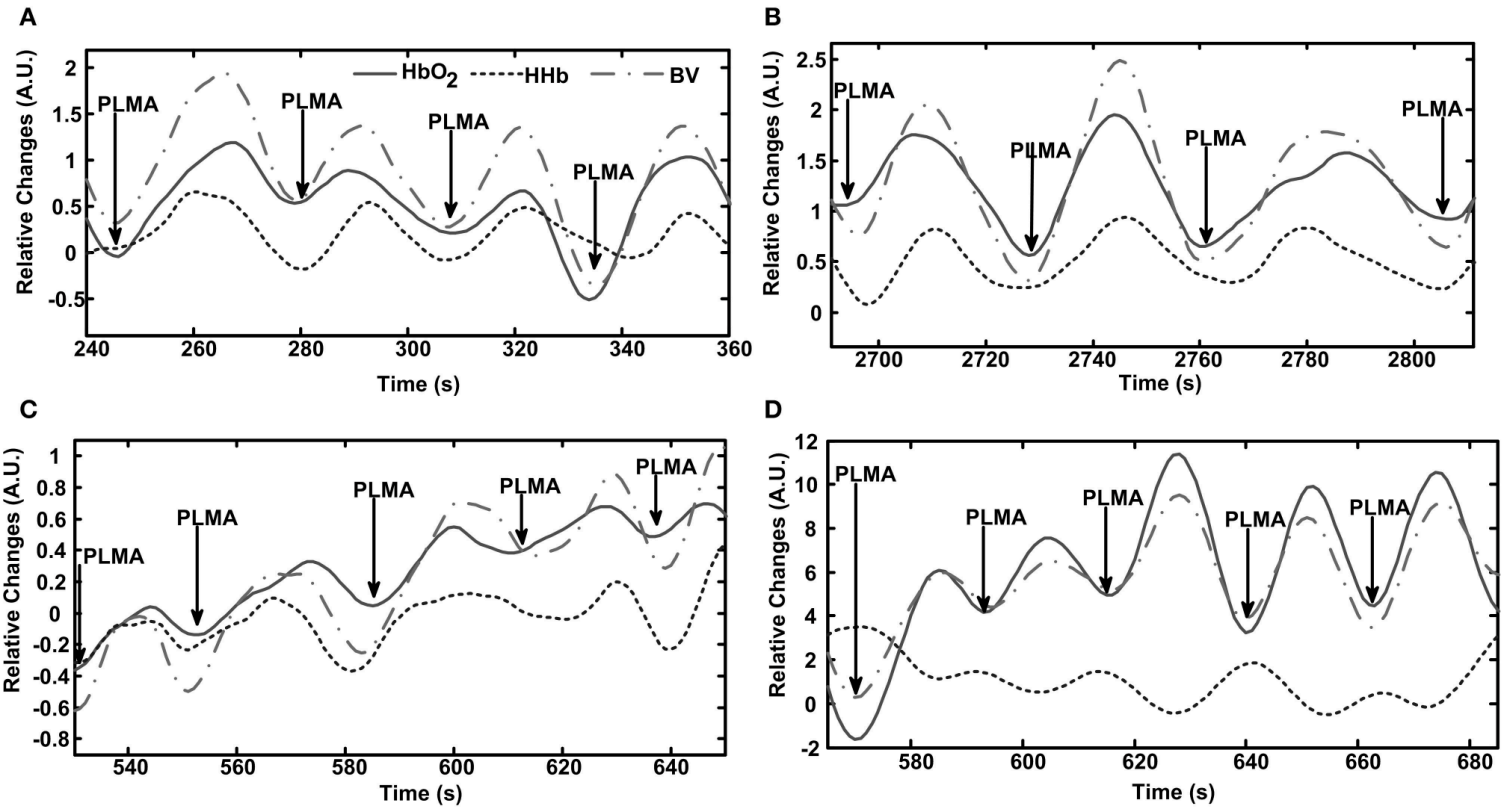
**Patterns of cerebral hemodynamic changes during periodic limb movement with arousal (PLMA) in each patient with PLMS**. A time window including several PLMA events is chosen to better illustrate the hemodynamic patterns. Subfigures **(A–D)** present results from Patient No. 1–4, respectively. PLMA events induce periodic changes in HbO_2_, HHb, and blood volume (BV), as shown in the panel of each subfigure. The hemodynamic changes are expressed in arbitrary units (A.U.), as the mean value of 10-s baseline measurements before the onset of the first event of the selected data fragment is set at 0.

**Figure 2:**
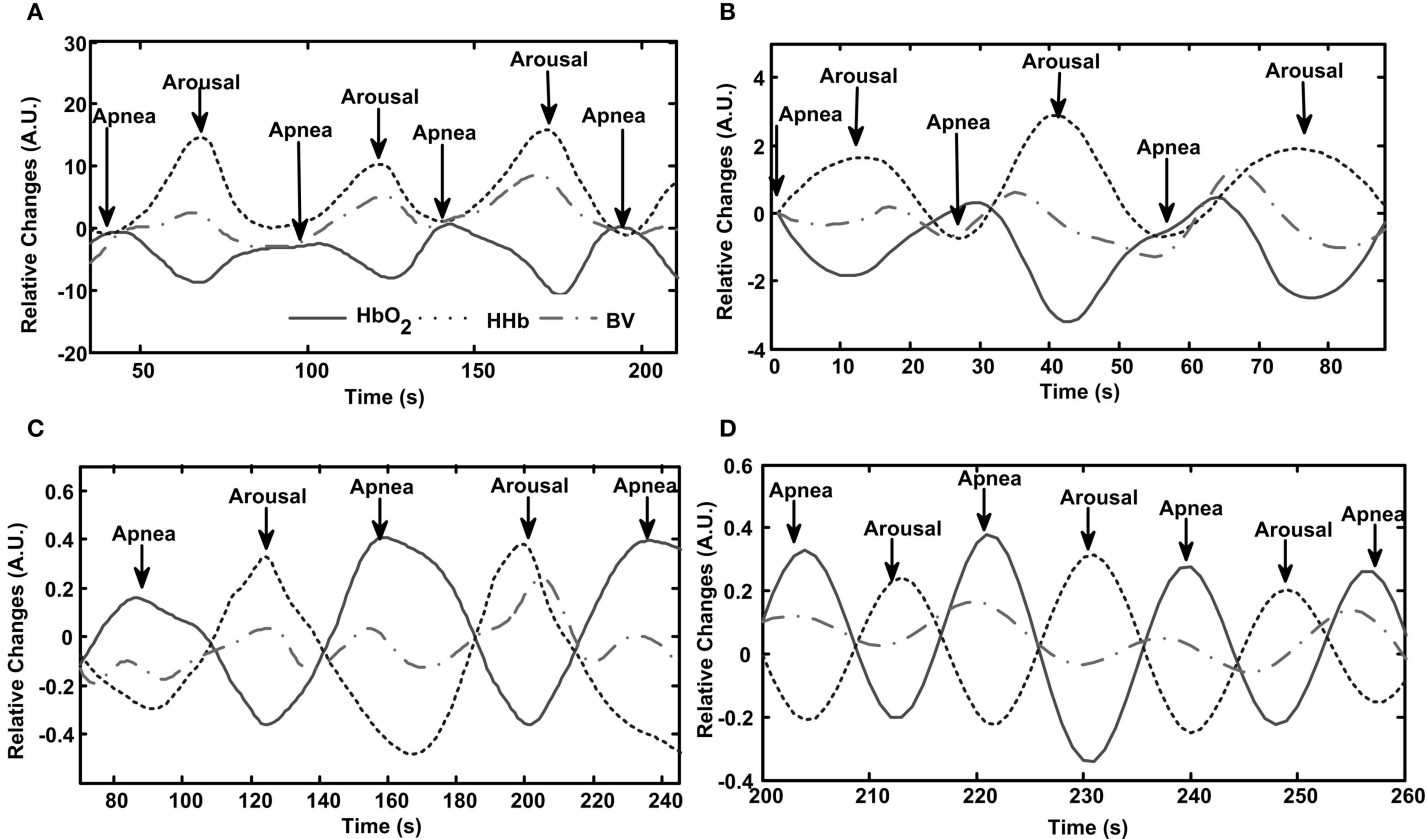
**Patterns of cerebral hemodynamic changes during apnea/hypopnea with arousal (AHA) in each patient with OSA**. Subfigures **(A–D)** present results from Patient No. 5–8, respectively. Repetitive AHA events induce periodic changes in HbO_2_, HHb and blood volume (BV), as shown in the panel of each subfigure.

Table 2 shows the length and number of valid AHA and PLMA events recorded from each patient. Totally we record 432 AHA and 459 PLMA events. Patterns of cerebral hemodynamic changes of each patient are illustrated in Figure 1 (Patient No. 1–4 with PLMS) and Figure 2 (Patient No. 5–8 with OSA).

**Table 2:** **The length and number of periodic AHA and PLMA events in all the patients**.

**Patient No**.	**Length of AHA (min)**	**No. of AHA events**	**Length of PLMA (min)**	**No. of PLMA events**
1	–	–	19	35
2	–	–	141	290
3	–	–	40	78
4	–	–	23.5	56
5	15	25	–	–
6	14	27	–	–
7	110	180	–	–
8	93	200	–	–
Total	232	432	223.5	459

*AHA, apnea/hypopnea with arousal; PLMA, periodic limb movement with arousal*.

As shown in Figure 1, PLMA events induce oscillations in HbO_2_, BV, and HHb in all the patients with PLMS, with periodicity approximates between 20 and 50 s. In Patient No. 1–3 (Figures 1A–C) HbO_2_, BV, and HHb repeatedly increase with the occurrence of each PLMA event and then decrease after reaching their peaks. In Patient No. 4 (Figure 1D) this pattern can only be found in HbO_2_ and BV, while HHb shows a reversing pattern.

Similarly, periodic AHA events in patients with OSA also induce AHA-related oscillations in cerebral hemodynamics, as illustrated in Figure 2. The oscillations induced by AHA are generally with comparable periodicities (20–60 s) as the ones induced by PLMA as shown in Figure 1. But the hemodynamic patterns are totally different between these two sleep disorders. In all the patients with OSA (Figures 2A–D), HbO_2_ decreases while HHb increases after the occurrence of AH, and these changing patterns reverse after the start of cortical arousals. The amplitudes of BV changes are relatively smaller compared to the ones of HbO_2_ and HHb because they are calculated as the sum of HbO_2_ and HHb. In Patient No. 5–7 (Figures 2A–C) we find a mild first increment and later decrement in BV during AHA events similar to the pattern of HHb. In Patient No. 8 (Figure 2D) we find the changing pattern of BV is similar as the one of HbO_2_.

### Area under the curve (AUC) changes of cerebral hemodynamics in apnea/hypopnea with arousal (AHA) and periodic limb movement with arousal (PLMA)

Table 3 below summarizes the descriptive statistics of AUC of HbO_2_, BV, and HHb during all the AHA and PLMA events in each patient. The changing patterns of hemodynamic parameters in PLMS and OSA mentioned above are confirmed by statistical comparisons (non-parametric Wilcoxon signed-rank test) of AUC changes to zero in each patient (*p* < 0.001 in most of the comparisons, with *p* = 0.005 in AUC of HHb in Patient No. 1, *p* = 0.028 in AUC of BV in Patient No. 5 and *p* = 0.012 in AUC of BV in Patient No. 6) except for the AUC of HHb in Patient No. 4 (*p* = 0.1), i.e., significantly positive values of AUC indicate a first increasing and later decreasing pattern, while negative AUC values suggest a first decreasing and later increasing pattern. On the group level, the Wilcoxon signed-rank test shows the AUC values are different from zero (*p* < 0.001). In Patient No. 4 the standard error of HHb AUC is about twice larger than the absolute value of mean (i.e., standard deviation is about 13 times larger than the mean with a sample size of 56 PLMA events) as shown in Table 3, which indicates overdispersion of the AUC and HHb changes may be heterogeneous. Therefore, we further divide the HHb changes in Patient No. 4 into two subgroups (i.e., groups with positive/negative AUC of HHb), and we find increasing HHb changes in 18 PLMA events and decreasing HHb in 38 ones. The bidirectional changes of HHb during PLMA events can also be found in other patients with PLMA, as shown in Table 4 below. Totally we find about 80% (367 out of 459) of PLMA events induce increasing HHb changes in all the patients.

**Table 3:** **Descriptive statistics of the area under the curve (AUC) of hemodynamic parameters in all the patients**.

	**Patient No**.	**AUC of HbO_2_**	**AUC of BV**	**AUC of HHb**
PLMS	1	13.11 (1.98)	15.65 (2.42)	1.69 (0.56)
	2	13.91 (1.39)	19.49 (1.77)	4.97 (0.37)
	3	4.60 (0.86)	7.96 (1.24)	3.11 (0.45)
	4	66.16 (12.12)	88.97 (9.6)	−5.63 (10.03)
	Total	18.64 (1.84)	25.72 (1.94)	3.11 (1.16)
OSA	5	−203.41 (35.06)	75.11 (31.39)	274.92 (56.30)
	6	−16.61 (4.35)	12.05 (5.81)	31.34 (4.55)
	7	−6.52 (0.67)	3.61 (0.28)	10.31 (0.73)
	8	−5.33 (0.38)	−1.40 (0.17)	3.08 (0.23)
	Total	−18.02 (3.05)	5.96 (2.04)	23.59 (4.47)

**Table 4:** **The area under the curve (AUC) of HHb with increasing and decreasing trends after the onset of periodic limb movement with arousal (PLMA) in all the patients**.

**Patient No**.	**AUC of increasing HHb**	**AUC of decreasing HHb**
1	3.34 (0.55), *n* = 23	−1.47 (0.31), *n* = 12
2	5.81 (0.40), *n* = 256	−1.33 (0.27), *n* = 34
3	3.61 (0.48), *n* = 70	−1.27 (0.24), *n* = 8
4	72.22 (16.50), *n* = 18	−42.51 (4.35), *n* = 38

*n is the number of PLMA events*.

### Heart rate (HR) changes in apnea/hypopnea with arousal (AHA) and periodic limb movement with arousal (PLMA)

**Figure 3:**
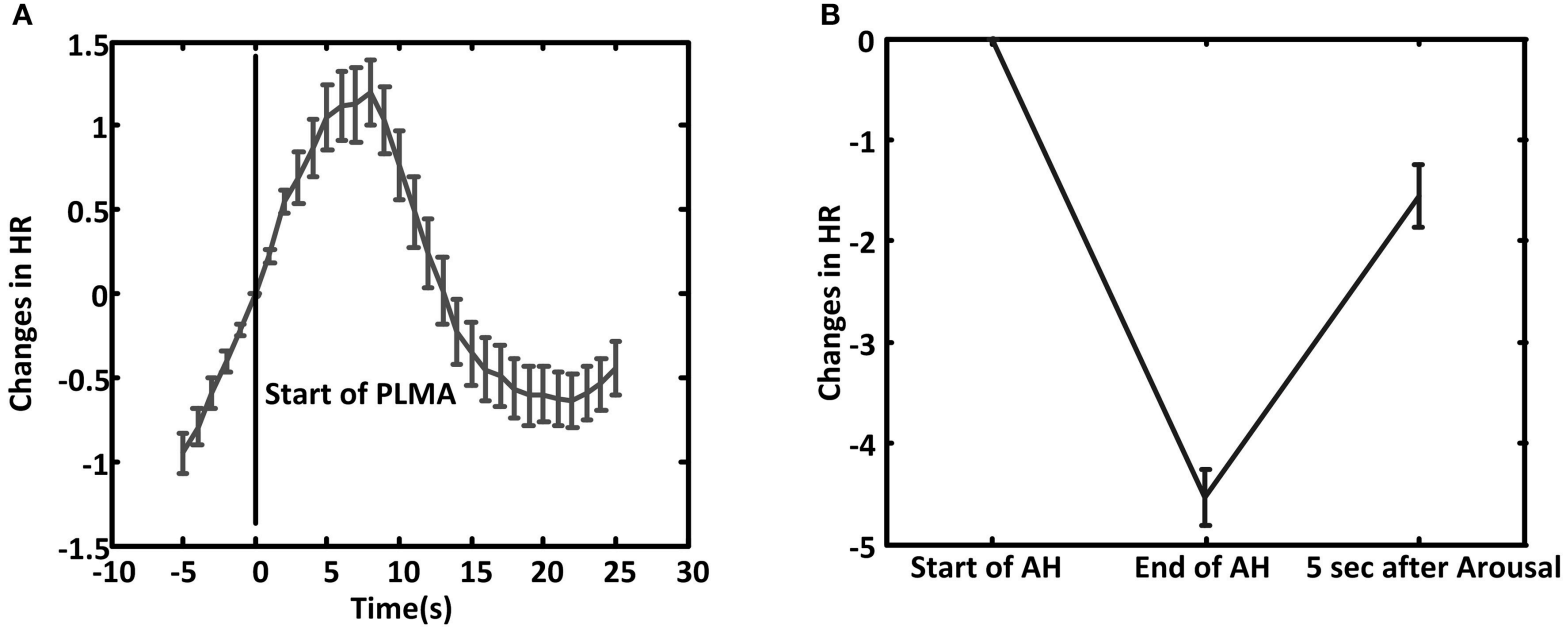
**The average of relative heart rate (HR) changes during PLMA (A) and AHA (B)**. The HR at the start of PLMA and apnea/hypopnea (AH) are set as reference value 0. Error bar is standard error.

In PLMA HR starts to increase before the onset of PLMA (Wilcoxon signed-rank test gives *p* < 0.001 by comparing the relative HR at 5 s before PLMA and at the onset of PLMA), as shown in Figure 3A. The increment reaches to peak in 5–8 s after the onset of PLMA, and then HR declines. Wilcoxon signed-rank test finds that the changes of HR are higher than the reference value 0 (*p* < 0.05) within 12 s after the onset of PLMA and there are no differences between HR at time 0 and 12–14 s, i.e., HR returns to its baseline value in 12–14 s after PLMA. After that HR continues decreasing to levels below baseline.

In AHA, HR decreases during AH (Wilcoxon signed-rank test gives *p* < 0.001 between HR at the onset of AH and the end of AH), as shown in Figure 3B. HR increases after the occurrence of arousal (Wilcoxon signed-rank test, *p* < 0.001).

## Discussion

In this preliminary study, our results of different cerebral hemodynamic patterns in PLMA and AHA challenge the hypothesis of common generator of these two events and point to different underlying mechanisms. HR shows a distinct pattern as it decreases during AH and increases with resumption of respiration, while in PLMA HR first increases and then decreases. We therefore propose that HR changes may partly account for cerebral BV changes in PLMA, but not for the ones in AHA. The differences between cerebral hemodynamics and HR changes induced by AHA and PLMA probably indicate different autonomic neural activations.

### Cerebral hemodynamic patterns in apnea/hypopnea with arousal (AHA) and periodic limb movement with arousal (PLMA)

Increase in BV during OSA has also been reported (Pizza et al., 2010; Ulrich et al., 2014). The increasing BV may indicate increasing blood flow mediated by several potential mechanisms such as hypercapnia/hypoxia induced cerebral vasodilatation, increased blood pressure, and sympathetic activity or cerebral vascular autoregulation (Jennum and Borgesen, 1989; Hayakawa et al., 1996; Somers, 2005; Olopade et al., 2007; Matsuo et al., 2011). Remarkably, in the patient with very severe OSA (a rare case with AHI value of 134/h), we find decreasing BV during AH events. The pathological mechanisms underlying such a decreasing BV are not clear. We speculate that aforementioned mechanisms are impaired and prevent the increase of blood flow during AH or alternatively other pathological mechanisms regulate the decreasing BV. Further studies are warranted because underlying pathophysiology may explain why patients with severe OSA are at high risk of detrimental cerebral vascular diseases, including stroke.

Only one other study published data on the cerebral hemodynamic changes induced by PLMS and found an increase in HbO_2_ and small decrease in HHb during PLMA events (Pizza et al., 2009). The increments in HbO_2_ and BV induced by PLMA may be due to increasing autonomic neural activation which is in line with previous studies demonstrating blood pressure and HR increase during PLMA (Ferrillo et al., 2004; Ferri et al., 2007; Siddiqui et al., 2007; Ferri and Zucconi, 2008; Pizza et al., 2009; Pennestri et al., 2013). Different from the invariable decrease in HHb in that study (Pizza et al., 2009), we find more heterogeneous increases or decreases of HHb during PLMA. This discrepancy is probably due to differences between the patients in these two studies. Although both studies are limited by a small sample size, the patients in Pizza et al. (2009) are more heterogeneous in respect to various cardiovascular comorbidities. Increasing HHb may suggest stronger oxygen extraction and cerebral oxygenation under the condition of increased HbO_2_ and BV. Whether the stronger cerebral oxygenation induced by nocturnal PLMA event could potentially account for the daytime sleepiness or fatigue in patients with PLMS will be an interesting hypothesis for the scientists in the relevant fields and warrants further investigations with large number of patients.

Our comparisons of hemodynamic changes following AHA and PLMA may provide valuable insights into the pathophysiology underlying both disorders. The different changes in cerebral BV may suggest that PLMA and AHA are regulated by different nervous systems. We can reasonably exclude hypercapnia/hypoxia induced vasodilatation as a mediating factor for increased BV in PLMA because the patients had normal ventilation during PLMA. More likely increased HR after the onset of PLMA may increase blood supply to the brain thus resulting in increments in cerebral HbO_2_, BV and HHb. This explanation is plausible considering that the HR changes a few seconds earlier than the onset of PLMA. Although we are cautious to interpret the temporal relationship between two events as a causal relationship, we may speculate about the possibility of some unknown master pacemaker governing both cerebral hemodynamics and HR changes in PLMA. The cerebral hemodynamics in AHA may not be well-interpreted by the autonomic branch that controlling HR variability alone, because the changes of cerebral BV conflict with the HR changes in most of our patients. Hypoxia or hypercapnia related changes in vasodilatation as well as other autonomic branches such as blood pressure may contribute to cerebral hemodynamic changes (Somers et al., 1995).

### HR changes in periodic limb movement with arousal (PLMA)

Although our findings of HR changes associated with PLMA fit the results of previous studies (Ferrillo et al., 2004; Ferri et al., 2007; Guggisberg et al., 2007; Ferri and Zucconi, 2008), the maximal increment of HR in our patients seems to be lower than the values previously reported (Ferri et al., 2007; Allena et al., 2009). This difference is probably due to the different selections of HR baseline and PLMA events. Previous studies preferred to analyze longer PLMA events (e.g., intervals of events are longer than 30 s (Ferri et al., 2007; Allena et al., 2009) and to choose HR a few seconds before the onset of PLMA as baseline that is supposed to avoid the potential confounding effects of the persisting HR changes from a leg movement preceding the one under analysis. In our study we do not set up such criteria to select the PLMA events because our primary interest is the NIRS signals after the onset and during the recovery phase of PLMA, rather than the quantification of HR changes. Therefore, we choose the HR at the onset of PLMA as baseline which is larger than the one in previous studies, because HR actually starts to increase a few second before the onset of leg movement. In addition, we do not distinguish unilateral and bilateral PLMA events, or PLMA in rapid eye movement (REM) and non-REM sleep either (Ferri and Zucconi, 2008). The mixture of different types of PLMA events may also partly explain the different maximal increments of HR after PLMA between our study and previous ones (Ferri and Zucconi, 2008). In fact, the maximal increment of HR after PLMA in our study is similar as the value reported by Guggisberg et al. who studied a mixture type of PLMA events without the selection of intervals (Guggisberg et al., 2007). Finally, another potential explanation is individual differences. The pathological mechanisms of PLMS in patients with different diseases like RLS, sleep apnea, narcolepsy or REM behavior disorder are quite probably to be different (Manconi et al., 2009). Our patients with PLMS all have RLS and OSA, so they may be a different phenotype of PLMS compared to the patients with RLS that are studied in previous studies (Ferri et al., 2007; Ferri and Zucconi, 2008).

### Limitations and future studies

The first limitation of our preliminary study is the limited number of patients in each group, but we still successfully record 432 AHA and 459 PLMA events. The second limitation is the relative low sample rate of our NIRS measurement (i.e., 1 Hz). Limb movement can be 0.5 s before or after start of the associated arousal, so future studies with higher NIRS sample rate may further clarify the exact start of hemodynamic changes in PLMA induced by limb movement or arousal. NIRS recordings with high sample rate can also allow us to extract pulse wave from the cortex during sleep (Mensen et al., 2016), thus pulse wave analysis may be adapted to analyze the NIRS-derived pulse wave in PLMA and AHA. Combining pulse wave analysis and changes in cerebral hemodynamics will open a new avenue to investigate neurovascular diseases like OSA, hypertension, and stroke (Cohn et al., 1995).

The third limitation may come from the superficial contribution to NIRS signals. Previous studies have found that BV measurement with NIRO-300 based on spatially resolved spectroscopy (SRS) algorithm can effectively reject interference by superficial tissues while BV measurement with MBLL should be interpreted with caution (Canova et al., 2011; Messere and Roatta, 2013). This is a crucial issue in NIRS studies especially when BV measured by these two methods shows inconsistency because that may suggest the measured changes of HbO_2_, HHb, and BV based on MBLL may actually come from superficial tissues rather than the brain (Canova et al., 2011). Several previous studies with different NIRS systems also found that cerebral BV and HbO_2_ changes calculated with MBLL contain superficial contribution, but HHb may be not significantly influenced (Kirilina et al., 2012; Hirasawa et al., 2015). In our preliminary data analysis, we found BV measured by SRS and MBLL actually show the same changing pattern in both AHA and PLMA events (see Supplementary Image 1), although the amplitude of BV calculated from SRS is smaller than the one measured by MBLL (i.e., BV calculated with MBLL includes additional contribution from superficial tissue). This indicates that the cerebral BV and superficial BV may show similar changing patterns in AHA and PLMA events. Considering that the source-detector distance of NIRO-300 in our study is 4 cm [which may mitigate the superficial contamination (Hirasawa et al., 2015)] and HHb changes may be not significantly influenced by superficial tissue, we think that the superficial contribution may overestimate the NIRS changes but the conclusions of this study should still hold. This is supported by our data of changes in HR and cerebral BV. If we mainly measured skin perfusion that manipulated by HR changes, then we would expect the same changes in BV after cortical arousals in AHA and PLMA because HR increases in both cases. But we find BV changes in different directions. Therefore, the measured BV in our study may mainly reflect cerebral physiology. This speculation is also supported by the fact that we found the same hemodynamic changing patterns as reported in previous NIRS studies with frequency domain NIRS (Pizza et al., 2010). Future NIRS studies applying multi-layered modeling approaches (which can better model the light transport through the head) may provide a solution to account for superficial effects.

Based on this preliminary study, several interesting questions will be raised to be further studied. New research topics in the relevant fields such as to explore the cerebral hemodynamics corresponding to pure cortical arousals in patients, PLM without cortical arousals or AH with arousals and respiratory-related leg movements, will further deepen our understanding of the pathophysiological mechanisms of PLMS and OSA. To combine muscular and cerebral NIRS hemodynamic recordings in patients with PLMS could provide an ideal methodology to study a current hypothesis of a potential peripheral trigger mechanism for PLMS (Salminen et al., 2013).

## Author contributions

ZZ: study design, data acquisition, analysis, and interpretation, manuscript preparation; MS: data acquisition, patients recruitment, and analysis. ML: data analysis and interpretation; MQ: patients recruitment and data acquisition; RK: study design, patients recruitment, analysis, and interpretation, manuscript preparation.

## Funding

This work was supported by Clinic Barmelweid Scientific Foundation and the Research Fund of the Swiss Lung Association No. 2014–22.

### Conflict of interest statement

The authors declare that the research was conducted in the absence of any commercial or financial relationships that could be construed as a potential conflict of interest.

## References

[B1] Afzelius L. E. (1981). Obstructive sleep apnea. N. Engl. J. Med. 305, 1472. 10.1056/NEJM198112103052413 7300868

[B2] Allena M. Campus C. Morrone E. De Carli F. Garbarino S. Manfredi C.. (2009). Periodic limb movements both in non-REM and REM sleep: relationships between cerebral and autonomic activities. Clin. Neurophysiol. 120, 1282–1290. 10.1016/j.clinph.2009.04.021 19505849

[B3] Al-Rawi P. G. Smielewski P. Kirkpatrick P. J. (2001). Evaluation of a near-infrared spectrometer (NIRO 300) for the detection of intracranial oxygenation changes in the adult head. Stroke 32, 2492–2500. 10.1161/hs1101.098356 11692006

[B4] Andreas S. Hajak G. von Breska B. Rüther E. Kreuzer H. (1992). Changes in heart rate during obstructive sleep apnoea. Eur. Respir. J. 5, 853–857. 1499710

[B5] Baran A. S. Richert A. C. Douglass A. B. May W. Ansarin K. (2003). Change in periodic limb movement index during treatment of obstructive sleep apnea with continuous positive airway pressure. Sleep 26, 717–720. 14572125 10.1093/sleep/26.6.717

[B6] Berry R. B. Budhiraja R. Gottlieb D. J. Gozal D. Iber C. Kapur V. K. (eds.) (2014). The AASM Manual for the Scoring of Sleep and Associated Events: Rules, Terminology and Technical Specifications. Darien, IL: American Academy of Sleep Medicine.

[B7] Canova D. Roatta S. Bosone D. Micieli G. (2011). Inconsistent detection of changes in cerebral blood volume by near infrared spectroscopy in standard clinical tests. J. Appl. Physiol. (1985) 110, 1646–1655. 10.1152/japplphysiol.00003.2011 21474700

[B8] Carelli G. Krieger J. Calvi-Gries F. Macher J. P. (1999). Periodic limb movements and obstructive sleep apneas before and after continuous positive airway pressure treatment. J. Sleep Res. 8, 211–216. 10.1046/j.1365-2869.1999.00153.x 10476008

[B9] Cleveland W. S. Devlin S. J. (1988). Locally weighted regression - an approach to regression-analysis by local fitting. J. Am. Stat. Assoc. 83, 596–610. 10.1080/01621459.1988.10478639

[B10] Cohn J. N. Finkelstein S. McVeigh G. Morgan D. LeMay L. Robinson J.. (1995). Noninvasive pulse wave analysis for the early detection of vascular disease. Hypertension 26, 503–508. 10.1161/01.HYP.26.3.503 7649589

[B11] Cuellar N. G. (2013). The effects of periodic limb movements in sleep (PLMS) on cardiovascular disease. Heart Lung 42, 353–360. 10.1016/j.hrtlng.2013.07.006 23998383

[B12] Ferri R. Rundo F. Zucconi M. Manconi M. Bruni O. Ferini-Strambi L.. (2015). An evidence-based analysis of the association between periodic leg movements during sleep and arousals in restless legs syndrome. Sleep 38, 919–924. 10.5665/sleep.4740 25581922 PMC4434558

[B13] Ferri R. Zucconi M. (2008). Heart rate and spectral EEG changes accompanying periodic and isolated leg movements during sleep. Sleep 31, 16–17. discussion: 18–19. 18220074 10.1093/sleep/31.1.16 PMC2225557

[B14] Ferri R. Zucconi M. Rundo F. Spruyt K. Manconi M. Ferini-Strambi L. (2007). Heart rate and spectral EEG changes accompanying periodic and non-periodic leg movements during sleep. Clin. Neurophysiol. 118, 438–448. 10.1016/j.clinph.2006.10.007 17140849

[B15] Ferrillo F. Beelke M. Canovaro P. Watanabe T. Aricó D. Rizzo P.. (2004). Changes in cerebral and autonomic activity heralding periodic limb movements in sleep. Sleep Med. 5, 407–412. 10.1016/j.sleep.2004.01.008 15223001

[B16] Guggisberg A. G. Hess C. W. Mathis J. (2007). The significance of the sympathetic nervous system in the pathophysiology of periodic leg movements in sleep. Sleep 30, 755–766. 17580597 10.1093/sleep/30.6.755 PMC1978348

[B17] Hayakawa T. Terashima M. Kayukawa Y. Ohta T. Okada T. (1996). Changes in cerebral oxygenation and hemodynamics during obstructive sleep apneas. Chest 109, 916–921. 10.1378/chest.109.4.916 8635370

[B18] Hirano Y. Stefanovic B. Silva A. C. (2011). Spatiotemporal evolution of the functional magnetic resonance imaging response to ultrashort stimuli. J. Neurosci. 31, 1440–1447. 10.1523/JNEUROSCI.3986-10.2011 21273428 PMC3078723

[B19] Hirasawa A. Yanagisawa S. Tanaka N. Funane T. Kiguchi M. Sørensen H.. (2015). Influence of skin blood flow and source-detector distance on near-infrared spectroscopy-determined cerebral oxygenation in humans. Clin. Physiol. Funct. Imaging 35, 237–244. 10.1111/cpf.12156 24750947

[B20] Hoshi Y. Mizukami S. Tamura M. (1994). Dynamic features of hemodynamic and metabolic changes in the human brain during all-night sleep as revealed by near-infrared spectroscopy. Brain Res. 652, 257–262. 10.1016/0006-8993(94)90235-6 7953738

[B21] Jennum P. Borgesen S. E. (1989). Intracranial pressure and obstructive sleep apnea. Chest 95, 279–283. 10.1378/chest.95.2.279 2914475

[B22] Kirilina E. Jelzow A. Heine A. Niessing M. Wabnitz H. Brühl R.. (2012). The physiological origin of task-evoked systemic artefacts in functional near infrared spectroscopy. Neuroimage 61, 70–81. 10.1016/j.neuroimage.2012.02.074 22426347 PMC3348501

[B23] Kohler M. Stradling J. R. (2010). Mechanisms of vascular damage in obstructive sleep apnea. Nat. Rev. Cardiol. 7, 677–685. 10.1038/nrcardio.2010.145 21079639

[B24] Manconi M. Ferri R. Ferini-Strambi L. (2009). A commentary on “Periodic limb movements both in non-REM and in REM sleep: relationships between cerebral and autonomic activities” by Allena et al. Clinical Neurophysiol. 2009;120:1282-90. Clin. Neurophysiol. 120, 1994–1995; author reply 1995–1997. 10.1016/j.clinph.2009.08.022 19815455

[B25] Manconi M. Ferri R. Zucconi M. Bassetti C. L. Fulda S. Aricó D.. (2012). Dissociation of periodic leg movements from arousals in restless legs syndrome. Ann. Neurol. 71, 834–844. 10.1002/ana.23565 22718547

[B26] Manconi M. Zavalko I. Bassetti C. L. Colamartino E. Pons M. Ferri R. (2014). Respiratory-related leg movements and their relationship with periodic leg movements during sleep. Sleep 37, 497. 10.5665/sleep.3484 24587572 PMC3920315

[B27] Matsuo A. Inoue Y. Namba K. Chiba H. (2011). Changes in cerebral hemoglobin indices in obstructive sleep apnea syndrome with nasal continuous positive airway pressure treatment. Sleep Breath 15, 487–492. 10.1007/s11325-010-0367-y 20589535

[B28] Mensen A. Zhang Z. Qi M. Khatami R. (2016). The occurrence of individual slow waves in sleep is predicted by heart rate. Sci. Rep. 6:29671. 10.1038/srep29671 27445083 PMC4957222

[B29] Messere A. Roatta S. (2013). Influence of cutaneous and muscular circulation on spatially resolved versus standard Beer-Lambert near-infrared spectroscopy. Physiol. Rep. 1:e00179. 10.1002/phy2.179 24744858 PMC3970749

[B30] Olopade C. O. Mensah E. Gupta R. Huo D. Picchietti D. L. Gratton E.. (2007). Noninvasive determination of brain tissue oxygenation during sleep in obstructive sleep apnea: a near-infrared spectroscopic approach. Sleep 30, 1747–1755. 18246984 10.1093/sleep/30.12.1747 PMC2276122

[B31] Pennestri M. H. Montplaisir J. Fradette L. Lavigne G. Colombo R. Lanfranchi P. A. (2013). Blood pressure changes associated with periodic leg movements during sleep in healthy subjects. Sleep Med. 14, 555–561. 10.1016/j.sleep.2013.02.005 23643655

[B32] Pierro M. L. Sassaroli A. Bergethon P. R. Ehrenberg B. L. Fantini S. (2012). Phase-amplitude investigation of spontaneous low-frequency oscillations of cerebral hemodynamics with near-infrared spectroscopy: a sleep study in human subjects. Neuroimage 63, 1571–1584. 10.1016/j.neuroimage.2012.07.015 22820416 PMC3472105

[B33] Pizza F. Biallas M. Wolf M. Valko P. O. Bassetti C. L. (2009). Periodic leg movements during sleep and cerebral hemodynamic changes detected by NIRS. Clin. Neurophysiol. 120, 1329–1334. 10.1016/j.clinph.2009.05.009 19540159

[B34] Pizza F. Biallas M. Wolf M. Werth E. Bassetti C. L. (2010). Nocturnal cerebral hemodynamics in snorers and in patients with obstructive sleep apnea: a near-infrared spectroscopy study. Sleep 33, 205–210. 20175404 10.1093/sleep/33.2.205 PMC2817907

[B35] Salminen A. V. Manconi M. Rimpilä V. Luoto T. M. Koskinen E. Ferri R.. (2013). Disconnection between periodic leg movements and cortical arousals in spinal cord injury. J. Clin. Sleep Med. 9, 1207–1209. 10.5664/jcsm.3174 24235905 PMC3805809

[B36] Scholkmann F. Kleiser S. Metz A. J. Zimmermann R. Mata Pavia J. Wolf U.. (2014). A review on continuous wave functional near-infrared spectroscopy and imaging instrumentation and methodology. Neuroimage 85(Pt 1), 6–27. 10.1016/j.neuroimage.2013.05.004 23684868

[B37] Scholkmann F. Wolf M. (2013). General equation for the differential pathlength factor of the frontal human head depending on wavelength and age. J. Biomed. Opt. 18:105004. 10.1117/1.JBO.18.10.105004 24121731

[B38] Seo W. H. Guilleminault C. (2012). Periodic leg movement, nasal CPAP, and expiratory muscles. Chest 142, 111–118. 10.1378/chest.11-1563 22241760

[B39] Sharma S. (2013). Obstructive sleep apnea and coronary artery pathology. Clin. Cardiol. 36, 300–301. 10.1002/clc.22119 23670853 PMC6649534

[B40] Siddiqui F. Strus J. Ming X. Lee I. A. Chokroverty S. Walters A. S. (2007). Rise of blood pressure with periodic limb movements in sleep and wakefulness. Clin. Neurophysiol. 118, 1923–1930. 10.1016/j.clinph.2007.05.006 17588809

[B41] Somers V. K. (2005). Sleep–a new cardiovascular frontier. N. Engl. J. Med. 353, 2070–2073. 10.1056/NEJMe058229 16282183

[B42] Somers V. K. Dyken M. E. Clary M. P. Abboud F. M. (1995). Sympathetic neural mechanisms in obstructive sleep apnea. J. Clin. Invest. 96, 1897–1904. 10.1172/JCI118235 7560081 PMC185826

[B43] Strangman G. Franceschini M. A. Boas D. A. (2003). Factors affecting the accuracy of near-infrared spectroscopy concentration calculations for focal changes in oxygenation parameters. Neuroimage 18, 865–879. 10.1016/S1053-8119(03)00021-1 12725763

[B44] Strollo P. J. J. Rogers R. M. (1996). Obstructive sleep apnea. N. Engl. J. Med. 334, 99–104. 10.1056/NEJM199601113340207 8531966

[B45] Suzuki S. Takasaki S. Ozaki T. Kobayashi Y. (1999). A tissue oxygenation monitor using NIR spatially resolved spectroscopy. Proc. SPIE 3597, 582–592. 10.1117/12.356862

[B46] Ulrich S. Nussbaumer-Ochsner Y. Vasic I. Hasler E. Latshang T. D. Kohler M.. (2014). Cerebral oxygenation in patients with obstructive sleep apnea. Effects of hypoxia at altitude and of acetazolamide. Chest 146, 299–308. 10.1378/chest.13-2967 24811331

[B47] van der Zee P. Cope M. Arridge S. R. Essenpreis M. Potter L. A. Edwards A. D.. (1992). Experimentally measured optical pathlengths for the adult head, calf and forearm and the head of the newborn infant as a function of inter optode spacing. Adv. Exp. Med. Biol. 316, 143–153. 10.1007/978-1-4615-3404-4_17 1288074

[B48] Walters A. S. Rye D. B. (2009). Review of the relationship of restless legs syndrome and periodic limb movements in sleep to hypertension, heart disease, and stroke. Sleep 32, 589–597. 19480225 10.1093/sleep/32.5.589 PMC2675893

[B49] Yaggi H. K. Concato J. Kernan W. N. Lichtman J. H. Brass L. M. Mohsenin V. (2005). Obstructive sleep apnea as a risk factor for stroke and death. New Engl. J. Med. 353, 2034–2041. 10.1056/NEJMoa043104 16282178

[B50] Yang C. K. Jordan A. S. White D. P. Winkelman J. W. (2006). Heart rate response to respiratory events with or without leg movements. Sleep 29, 553–556. 16676789 10.1093/sleep/29.4.553

[B51] Zhai J. Li T. Zhang Z. Gong H. (2009). Hemodynamic and electrophysiological signals of conflict processing in the Chinese-character Stroop task: a simultaneous near-infrared spectroscopy and event-related potential study. J. Biomed. Opt. 14:054022. 10.1117/1.3247152 19895124

[B52] Zhang Z. Khatami R. (2014). Predominant endothelial vasomotor activity during human sleep: a near-infrared spectroscopy study. Eur. J. Neurosci. 40, 3396–3404. 10.1111/ejn.12702 25156240

[B53] Zhang Z. Khatami R. (2015). A biphasic change of regional blood volume in the frontal cortex during non-rapid eye movement sleep: a near-infrared spectroscopy study. Sleep 38, 1211–1217. 10.5665/sleep.4894 25761983 PMC4507726

[B54] Zhang Z. Schneider M. Fritschi U. Lehner I. Khatami R. (2014). Near-infrared spectroscopy (NIRS) as a useful tool to evaluate the treatment efficacy of positive airways pressure therapy in patients with obstructive sleep apnea syndrome (OSAS): a pilot study. J. Innov. Opt. Health Sci. 7:1450014 10.1142/S179354581450014X

[B55] Zucconi M. Ferri R. Allen R. Baier P. C. Bruni O. Chokroverty S.. (2006). The official World Association of Sleep Medicine (WASM) standards for recording and scoring periodic leg movements in sleep (PLMS) and wakefulness (PLMW) developed in collaboration with a task force from the International Restless Legs Syndrome Study Group (IRLSSG). Sleep Med. 7, 175–183. 10.1016/j.sleep.2006.01.001 16459136

