# Supplementary material for: The Comparisons of Cerebral Hemodynamics Induced by Obstructive Sleep Apnea with Arousal and Periodic Limb Movement with Arousal: A Pilot NIRS Study
Source: Front Neurosci. 2016 Aug 31;10:403. doi: 10.3389/fnins.2016.00403 (PMC5005379; doi:10.3389/fnins.2016.00403)
Supplement: Supplementary file 1 [file Image1.PDF]

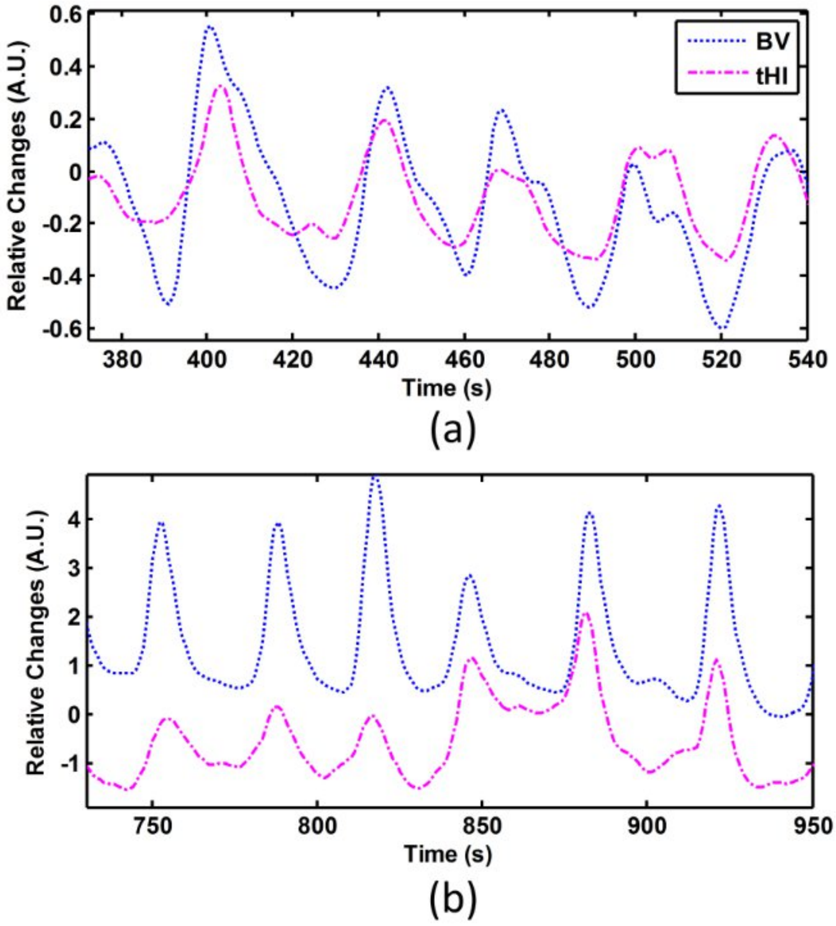


Figure. Example of blood volume changes measured from modified Beer-Lambert law (MBLL) and spatially resolved spectroscopy (SRS) in PLMA (a) and AHA (b). BV indicated blood volume measured from MBLL, and tHI is blood volume measured from SRS.
